# NRP-1 silencing suppresses hepatocellular carcinoma cell growth *in vitro* and *in vivo*

**DOI:** 10.3892/etm.2012.803

**Published:** 2012-11-06

**Authors:** JING XU, JINGLIN XIA

**Affiliations:** 1 The Fifth People’s Hospital; 2 Liver Cancer Institute, Fudan University;; 3 Department of Hepatic Oncology, Zhongshan Hospital, Fudan University, Shanghai 200032, P.R. China

**Keywords:** hepatocellular carcinoma, tumor growth, microvessel density, neuropilin-1

## Abstract

Neuropilin-1 (NRP-1) is a novel receptor of vascular endothelial growth factor 165 that promotes angiogenesis, tumor growth, tumor invasion and metastasis. However, its role in tumorigenesis and progression of human hepatocellular carcinoma (HCC) is unknown. In this study, lentivirus-mediated short hairpin RNA (shRNA) was used to silence NRP-1 in the HCCLM6 cell line to explore its role in regulating the growth of HCC. Recombinant NRP-1 shRNA lentivirus was prepared and transfected into HCCLM6 cells. Transfection efficiencies of the lentivirus were observed by flow cytometry. Protein and mRNA expression of NRP-1 were examined by western blot analysis and quantitative reverse transcription-polymerase chain reaction (RT-PCR), and the effect of the lentivirus on cell growth was determined using MTT assay. Different cell groups were inoculated into nude mice to establish cancer xenografts, and tumor growth was monitored. Protein expression of NRP-1 in tumor tissues was detected by western blot assay. Microvessel density (MVD) in tumor tissues was assessed by immunohistochemistry (IHC). Lentivirus-mediated shRNA efficiently reduced endogenous NRP-1 expression in HCCLM6 cells and significantly inhibited cell growth *in vitro. In vivo*, NRP-1 knockdown in tumor tissues resulted in decreased vasculature. NRP-1 promotes the growth of HCC *in vitro* and *in vivo*, and therefore may be considered as a novel therapeutic target for HCC.

## Introduction

HCC is the third leading cause of cancer mortality in the world, and the second in China (1,2). Even with improvement in surgical procedures and other adjuvant therapies, rapid growth, high recurrence and metastasis are still the main cause of treatment failure and cancer mortalities. Angiogenesis is essential for the growth, invasion and metastasis of solid tumors. Therefore, anti-angiogenesis therapy may inhibit the development of HCC and prolong the lives of patients.

NRP-1 was first identified as a 120–130 kDa membrane protein from the optic tract of *Xenopus laevis*(3). It functions as a semaphorin (SEMA) receptor in the developing nervous system (4–6). Subsequently, NRP-1 was found to be a co-receptor for vascular endothelial growth factor 165 (VEGF 165) and is expressed in endothelial cells (EC), where it is involved in the regulation of angiogenesis and endothelial cell migration (7–9). Overexpression of NRP-1 in a transgenic mouse model increased capillary and blood vessel formation and resulted in hemorrhage (10), whereas functional inactivation of NRP-1 in mice led to embryonic lethality with multiple vascular abnormalities, including of avascular regions, heterogeneous blood vessel size and abnormally formed dorsal aorta (11). These results indicated that NRP-1 was a key regulator of developmental angiogenesis.

NRP-1 is also expressed in several tumors, including glioma, acute myeloid leukemia, pancreatic, lung, breast, prostate, colon and gastric cancers, where it regulates the growth, invasion and metastasis of malignant tumors (12–20). However, the role of NRP-1 in HCC progression remains unknown. In this study, the endogenous NRP-1 expression in HCCLM6 cells was knocked down using lentivirus-mediated RNA interference (RNAi), to investigate the role of NRP-1 in regulating HCC progression.

## Materials and methods

### Cell line and laboratory animals

The human hepatoma-derived cell line HCCLM6, with a high metastatic ability as evaluated by xenograft models, was established by the Liver Cancer Institute (Fudan University, Shanghai, China) and cultured in Dulbecco’s modified Eagle’s medium (DMEM) (Gibco BRL, Grand Island, NY, USA) supplemented with 10% fetal bovine serum (FBS). All cells were maintained at 37°C in a humidified incubator containing 5% CO_2_. Five-week-old male nude mice with an average weight of 20±5 g were purchased from Institute of Materia Medica (CAS, Shanghai, China). The nude mice were kept in specific pathogen-free conditions with lamina flow devices in accordance with related regulations. The study was approved by the ethics committee of Zhongshan Hospital (Fudan University, China).

### Construction and transfection of lentiviral vectors with specific shRNA for NRP-1

The RNAi candidate sequences for human NRP-1 were designed according to a previous study (13) and the detailed sequences were as follows: sense, 5′-GAGAGGTCCTGAATGTTCC-3′; anti-sense, 5′-GGAACATTCAGGACCTCTC-3′. Stem-loop DNA oligonucleotides were synthesized and cloned into the lentivirus-based RNAi vector pLVTHM. The negative control plasmid was formed by an empty vector pLVTHM. Lentiviral particles were prepared as described previously (21). HCCLM6 cells were seeded in six-well plates at a concentration of 5×10^5^ per well. Lentivirus transfection was conducted when the cells reached 70–80% confluence. Cells were divided into three groups as follows: the knockdown (KD) cells were transfected with NRP-1 shRNA lentivirus (MOI 30); the negative control (NC) cells were transfected with empty lentivirus (MOI 30) and the blank control (BC) cells were not transfected. After 48 h, transfection efficiency was detected using flow cytometry (BD Pharmingen, San Diego, CA, USA).

### Real-time PCR assay

Total RNA extraction was carried out using the TRIzol Reagent (Invitrogen, Carlsbad, CA, USA) according to the manufacturer’s instructions. RNA quality was assured by the A260/280 absorbance ratio and 0.5 *μ*g total RNA was reverse transcribed into single-strand cDNA using PrimeScript™ RT Enzyme Mix I (Takara, Kyoto, Japan). RT-PCR was carried out for 15 min at 37°C and 5 sec at 85°C in a thermocycler. For the NRP-1 gene, 2.0 *μ*l cDNA template was used for routine PCR in a final volume of 20 *μ*l. The forward primer 5′-TCCCGCCTGAACTACCCTTGAGA-3′ and the reverse primer 5′-TTTGAAATGGCGCCCTGTGTCC-3′ were used and amplified in one cycle at 95°C for 10 sec, then 40 cycles at 95°C for 5 sec and 60°C for 30 sec. To measure the relative amount of the NRP-1 gene in the different samples, the glyceraldehyde 3-phosphate dehydrogenase (GAPDH) level was determined and employed as a control. The PCR was performed in a DNA Engine Opticon system (MJ Research, Reno, NV, USA) by using SYBR^®^-Green PCR Master mix (Takara, Kyoto, Japan). The ΔΔCt method was used for relative quantitative comparison among samples (22).

### Western blot analysis

Approximately 20 mg total protein extracted from groups of cells were separated on 6% sodium dodecyl sulphate polyacrylamide gel (SDS-PAGE) and transferred onto polyvinylidene fluoride membranes (Millipore, MA, USA). After blocking the membranes, the diluted primary antibodies against NRP-1 and GAPDH (Santa Cruz Biotechnology, CA, USA) were incubated for 24 h at 4°C. After extensive washing in Tris-buffered saline (TBS) buffer, the membranes were incubated with horseradish peroxidase-conjugated goat anti-mouse secondary antibody for 1 h at room temperature. Labeled proteins on western blots were visualized using the Chemiluminescence Reagent Plus detection system (New England Nuclear, Boston, MA, USA). Quantification of bands was carried out using an Alpha Imager (Alpha Innotech, San Leandro, CA, USA).

### MTT analysis

Three groups of cells were individually seeded into 96-well plates at a concentration of 7×10^3^ per well filled with DMEM supplemented with 10% FBS. After 24 h, the medium was removed and replaced with fresh medium. One plate was examined immediately after the medium change and other plates were analyzed every 24 h for 3 days. Assays were initiated by adding 20 *μ*l MTT (5 mg/ml) to each well and incubating the cells for an additional 4 h. Finally, the medium was removed and 150 *μ*l dimethylsulphoxide (DMSO) was added to each well. Plates were read at a wavelength of 570 nm.

### In vivo tumor model

Eighteen nude mice were randomly divided into 3 groups and each was subcutaneously injected with 1×10^7^ KD cells, NC cells or BC cells, respectively, in the right upper flank region, for the establishment of subcutaneous xenograft models. Every 5 days after the injection, tumors in the mice were observed visually. Mice were sacrificed at 50 days and tumor mass and volume were recorded. Volume was calculated as (length/2) × (width^2^). Then NRP-1 protein expression in tumors was detected by western blot analysis as described above.

### Immunohistochemistry

One xenograft from each group was formalin-fixed, paraffin-embedded and sectioned into 4-*μ*m-thick sections. Then, sections were deparaffinized in xylene and treated with a graded series of alcohol [100, 95 and 80% (V/V) ethanol in double-distilled water] and rehydrated in phosphate-buffered saline (pH 7.5). For MVD assessment, the slides were microwaved for 5 min for antigen retrieval. Then rat monoclonal anti-CD34 antibody (Abcam, Cambridgeshire, UK) was added and incubated at room temperature for 2 h. Afterwards, horseradish peroxidase-conjugated goat anti-rat secondary antibody (Beyotime, Shanghai, China) was added and incubated for a further 1 h at room temperature. Slides were incubated with stable 3,3′-diaminobenzidine (DAB) for 10–15 min, and then rinsed with distilled water and counter-stained with Gill’s hematoxylin (Sigma, St. Louis, MO, USA) for 1 min. Then slides were observed under a Leica CTR 5000 microscope system (Leica Microsystems, Hong Kong, China) to count MVD as described previously (23).

### Statistical analysis

Statistical analysis was performed using SPSS 13.0 software (SPSS Inc., Chicago, IL, USA) using a Student’s t-test throughout the present study. P<0.05 was considered to indicate a statistically significant difference.

## Results

### NRP-1 shRNA lentivirus significantly suppressed mRNA and protein expression of NRP-1

**Figure 1:**
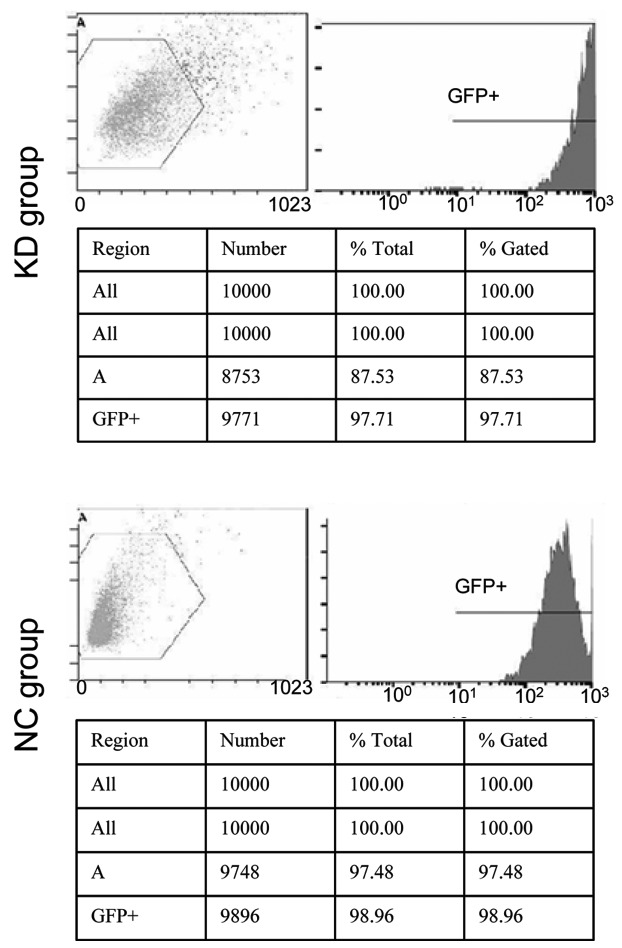
Transfection efficiencies of NRP-1 shRNA lentivirus (KD group) and empty lentivirus (NC group). Normalized by the HCCLM6 cells (BC group), GFP-positive cell percentage in the gate represented transfection efficiency. The two groups had high efficiency (>95%). NRP-1, neuropilin-1.

**Figure 2:**
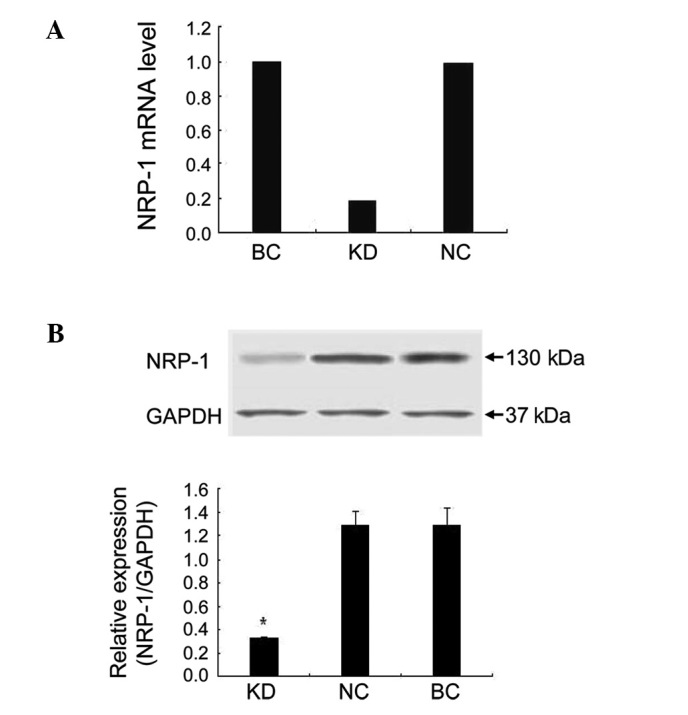
Knockdown of NRP-1 gene by NRP-1 shRNA lentivirus. (A) Real-time PCR showed a significant decrease of NRP-1 mRNA (by 81.5%) in the KD group vs. BC group. (B) Western blot assay demonstrated that, normalized by GAPDH, NRP-1 protein expression was degraded (by 74.9%) in the KD group vs. BC group with statistical significance, P<0.05. NRP-1, neuropilin-1; PCR, polymerase chain reaction; KD, knockdown; BC, blank control; NC, negative control; GAPDH, glyceraldehyde 3-phosphate dehydrogenase.

After 48 h transfection, green fluorescent protein (GFP) expression rates of the KD and NC cells were 97.71 and 98.96%, respectively (Fig. 1). The real-time PCR results demonstrated that NRP-1 shRNA had a significant suppressive effect. The NRP-1 mRNA level of the KD group decreased by 81.5% compared to the BC group. In the NC group cells transfected with empty lentivirus, the expression of NRP-1 mRNA was barely affected (Fig. 2A). In the western blot assay, NRP-1 protein expression was inhibited by 74.9% in the KD group compared to the BC group (P<0.05). No obvious variance was found between the NC and BC group (P>0.05; Fig. 2B).

### NRP-1 shRNA lentivirus led to inhibition of cell growth in vitro

**Figure 3:**
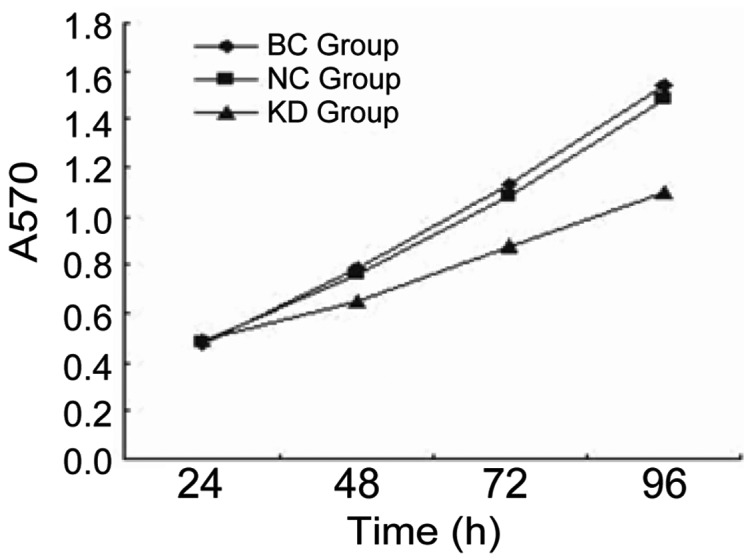
Cell growth rate of the KD, NC and BC group. As shown in the growth curve, cells transfected with NRP-1 shRNA lentivirus (KD group) grew more slowly than untransfected cells (BC group). Statistical analysis was performed by comparing cell growth rate of different groups at 48, 72 and 96 h, with a result of decreased growth rate in KD group vs. BC group at different times. KD, knockdown; NC, negative control; BC, blank control; NRP-1, neuropilin-1.

For the MTT assay, the growth rate of the KD group was significantly lower than that of the BC and NC groups (P<0.05). There was no significant difference between the NC and BC group (P>0.05; Fig. 3).

### NRP-1 shRNA lentivirus suppressed NRP-1 protein expression and tumor growth in xenografts

**Figure 4:**
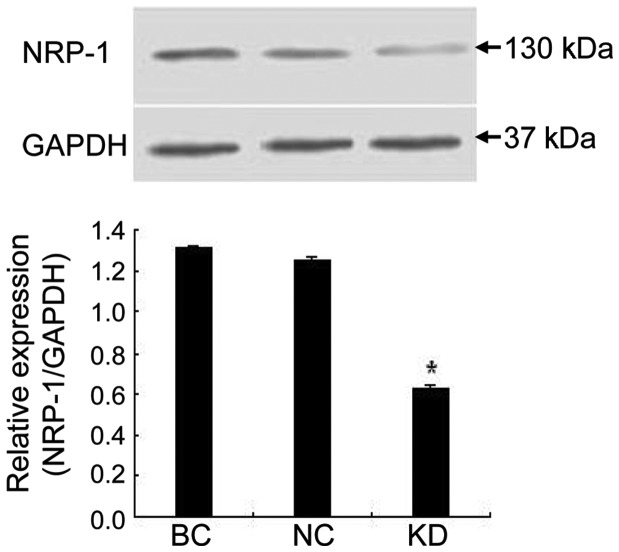
NRP-1 protein expression in xenograft tumors. Similar to the western blot assay *in vitro*, NRP-1 protein expression in xenograft tumors was degraded (by 52.2%) in the KD group vs. the BC group with statistical significance, P<0.05. NRP-1, neuropilin-1; KD, knockdown; BC, blank control; NC, negative control.

As shown in Fig. 4, NRP-1 shRNA also caused significant inhibition of NRP-1 protein expression in xenograft tumor tissue. NRP-1 protein expression was inhibited by 52.2% in the KD group compared to the BC group (P<0.05) while little variance was found between the NC and BC group (P>0.05). Additionally, we found that tumors in the KD group were smaller in size and lighter in weight than those in the BC group (P<0.05), while no obvious difference was found between the NC and BC group (P>0.05; Table I).

### NRP-1 shRNA lentivirus led to decreased angiogenesis in vivo

**Figure 5:**
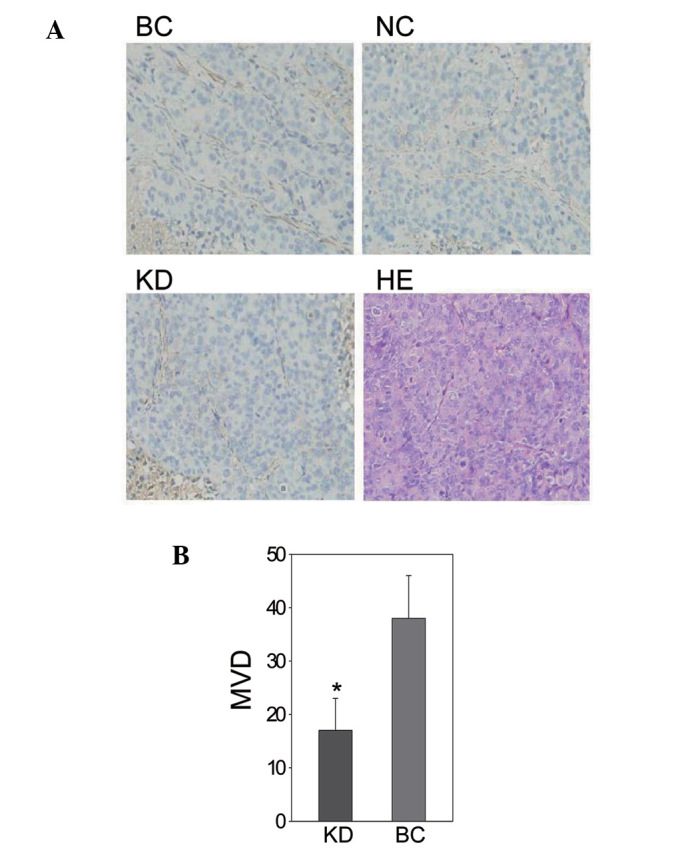
(A) Microvessel density (MVD) of xenograft tumors in the knockdown (KD), negative control (NC) and blank control (BC) groups (magnification, ×200). (B) NRP-1 silencing led to significantly decreased MVD in the KD group (17±6) vs. the BC group (38±8), P<0.05.

As the results of the immunohistochemical analysis showed, NRP-1 shRNA had a suppressive effect on the neovascularization and angiogenesis of tumors. The MVD of the KD group was 17±6, lower than that of the BC group (38±8; P<0.05) while no significant difference was found between the NC group (34±7) and the BC group (P>0.05; Fig. 5).

## Discussion

In this study, NRP-1 shRNA lentivirus caused inhibition of NRP-1 mRNA and protein expression, thus suppressing HCC cell growth *in vitro. In vivo*, it also downregulated NRP-1 protein expression in xenograft tumors, whose growth and angiogenesis were significantly suppressed. These findings demonstrated that NRP-1 played an important role in HCC growth by promoting neovascularization and angiogenesis.

Considerable experimental evidence has shown that NRP-1 plays an essential role in the tumor growth and metastasis by regulating angiogenesis. The majority of studies supported that NRP-1 functioned as a co-receptor of VEGF 165 and enhanced vascular endothelial growth factor receptor-2 (VEGFR-2) activity in the presence of VEGF (9,17,19,24,25). However, several papers studying tumor cells lacking VEGFR-2 expression have suggested that NRP-1 may transduce VEGF-mediated signals either alone or in concert with other receptors (13,15,26,27). Recently, NRP-1 was found to promote tumor progression by interacting with other proteins, such as integrin beta-1 and hepatocyte growth factor/scatter factor (18,20). However, the exact pathway mediated by NRP-1 in angiogenesis of HCC needed further investigation.

The NRP-1 shRNA lentivirus achieved an effective gene silence *in vitro*, as NRP-1 protein expression was decreased by 74.9% in transfected cells. However, in the xenograft tumors, NRP-1 shRNA only led to a 52.2% decrease of NRP-1 protein. Theoretically, NRP-1 shRNA should have the same gene silencing effect *in vivo* as *in vitro.* Many reasons may be responsible for the difference. One is that the tumor tissue removed from mice for protein extraction not only included HCC cells, but also vascular endothelial cells (ECs), mononuclear cells and so on, which may have normal NRP-1 protein expression.

HCC is a hypervascular tumor and a rich vascular bed providing the necessary nutrients for tumor growth and providing a channel for tumor invasion and metastasis. Knockdown of NRP-1 by the NRP-1 shRNA lentivirus led to a significant reduction of tumor MVD, significantly inhibiting the growth of HCC. In conclusion, NRP-1 is a potential target for anti-angiogenetic therapy for HCC.

## Figures and Tables

**Table I:** Xenograft tumors in the KD, NC and BC group.

Group	Volume (cm^3^)	P-value	Weight (g)	P-value
KD	1.34±0.12	0.000	1.39±0.18	0.000
NC	1.46±0.14	0.11	1.98±0.16	0.089
BC	1.43±0.12	-	1.96±0.13	-

KD, NRP-1 shRNA lentivirus (knockdown); NC, empty lentivirus (normal control); BC, normalized by the HCCLM6 cells (blank control).

## References

[1] Tang ZY (2001). Hepatocellular carcinoma-cause, treatment and metastasis. World J Gastroenterol.

[2] Parkin DM, Bray F, Ferlay J, Pisani P (2005). Global cancer statistics, 2002. CA Cancer J Clin.

[3] Fujisawa H, Takagi S, Hirata T (1995). Growth-associated expression of a membrane protein, neuropilin, in Xenopus optic nerve fibers. Dev Neurosci.

[4] Kolodkin AL, Levengood DV, Rowe EG, Tai YT, Giger RJ, Ginty DD (1997). Neuropilin is a semaphorin III receptor. Cell.

[5] He Z, Tessier-Lavigne M (1997). Neuropilin is a receptor for the axonal chemorepellent Semaphorin III. Cell.

[6] Rossignol M, Gagnon ML, Klagsbrun M (2000). Genomic organization of human neuropilin-1 and neuropilin-2 genes: identification and distribution of splice variants and soluble isoforms. Genomics.

[7] Bernatchez PN, Rollin S, Soker S, Sirois MG (2002). Relative effects of VEGF-A and VEGF-C on endothelial cell proliferation, migration and PAF synthesis: role of neuropilin-1. J Cell Biochem.

[8] Lee P, Goishi K, Davidson AJ, Mannix R, Zon L, Klagsbrun M (2002). Neuropilin-1 is required for vascular development and is a mediator of VEGF-dependent angiogenesis in zebrafish. Proc Natl Acad Sci U S A.

[9] Soker S, Takashima S, Miao HQ, Neufeld G, Klagsbrun M (1998). Neuropilin-1 is expressed by endothelial and tumor cells as an isoform-specific receptor for vascular endothelial growth factor. Cell.

[10] Kitsukawa T, Shimono A, Kawakami A, Kondoh H, Fujisawa H (1995). Overexpression of a membrane protein, neuropilin, in chimeric mice causes anomalies in the cardiovascular system, nervous system and limbs. Development.

[11] Takashima S, Kitakaze M, Asakura M (2002). Targeting of both mouse neuropilin-1 and neuropilin-2 genes severely impairs developmental yolk sac and embryonic angiogenesis. Proc Natl Acad Sci U S A.

[12] Hansel DE, Wilentz RE, Yeo CJ, Schulick RD, Montgomery E, Maitra A (2004). Expression of neuropilin-1 in high-grade dysplasia, invasive cancer, and metastases of the human gastrointestinal tract. Am J Surg Pathol.

[13] Bachelder RE, Lipscomb EA, Lin X (2003). Competing autocrine pathways involving alternative neuropilin-1 ligands regulate chemotaxis of carcinoma cells. Cancer Res.

[14] Stephenson JM, Banerjee S, Saxena NK, Cherian R, Banerjee SK (2002). Neuropilin-1 is differentially expressed in myoepithelial cells and vascular smooth muscle cells in preneoplastic and neoplastic human breast: a possible marker for the progression of breast cancer. Int J Cancer.

[15] Ochiumi T, Kitadai Y, Tanaka S, Akagi M, Yoshihara M, Chayama K (2006). Neuropilin-1 is involved in regulation of apoptosis and migration of human colon cancer. Int J Oncol.

[16] Kreuter M, Woelke K, Bieker R (2006). Correlation of neuropilin-1 overexpression to survival in acute myeloid leukemia. Leukemia.

[17] Lu L, Zhang L, Xiao Z, Lu S, Yang R, Han ZC (2008). Neuropilin-1 in acute myeloid leukemia: expression and role in proliferation and migration of leukemia cells. Leuk lymphoma.

[18] Hu B, Guo P, Bar-Joseph I (2007). Neuropilin-1 promotes human glioma progression through potentiating the activity of the HGF/SF autocrine pathway. Oncogene.

[19] Hong TM, Chen YL, Wu YY (2007). Targeting neuropilin 1 as an antitumor strategy in lung cancer. Clin Cancer Res.

[20] Fukasawa M, Matsushita A, Korc M (2007). Neuropilin-1 interacts with integrin beta1 and modulates pancreatic cancer cell growth, survival and invasion. Cancer Biol Ther.

[21] Lois C, Hong EJ, Pease S, Brown EJ, Baltimore D (2002). Germline transmission and tissue-specific expression of transgenes delivered by lentiviral vectors. Science.

[22] Livak KJ, Schmittgen TD (2001). Analysis of relative gene expression data using real-time quantitative PCR and the 2(-DeltaDeltaC(T)) Method. Methods.

[23] Weidner N, Semple JP, Welch WR, Folkman J (1991). Tumor angiogenesis and metastasis-correlation in invasive breast carcinoma. New Engl J Med.

[24] Miao HQ, Klagsbrun M (2000). Neuropilin is a mediator of angiogenesis. Cancer Metastasis Rev.

[25] Miao HQ, Lee P, Lin H, Soker S, Klagsbrun M (2000). Neuropilin-1 expression by tumor cells promotes tumor angiogenesis and progression. Faseb J.

[26] Bachelder RE, Crago A, Chung J (2001). Vascular endothelial growth factor is an autocrine survival factor for neuropilin-expressing breast carcinoma cells. Cancer Res.

[27] Barr MP, Byrne AM, Duffy AM (2005). A peptide corresponding to the neuropilin-1-binding site on VEGF(165) induces apoptosis of neuropilin-1-expressing breast tumour cells. Br J Cancer.

